# The Evolution and Characterization of the RNA Interference Pathways in Lophotrochozoa

**DOI:** 10.1093/gbe/evae098

**Published:** 2024-05-07

**Authors:** Alessandro Formaggioni, Gianmarco Cavalli, Mayuko Hamada, Tatsuya Sakamoto, Federico Plazzi, Marco Passamonti

**Affiliations:** Department of Biological, Geological and Environmental Sciences, University of Bologna, Bologna, Italy; Department of Biological, Geological and Environmental Sciences, University of Bologna, Bologna, Italy; Ushimado Marine Institute, Okayama University, Okayama, Japan; Ushimado Marine Institute, Okayama University, Okayama, Japan; Department of Biological, Geological and Environmental Sciences, University of Bologna, Bologna, Italy; Department of Biological, Geological and Environmental Sciences, University of Bologna, Bologna, Italy

**Keywords:** Metazoa, Lophotrochozoa, Argonaute, DICER, RNAi

## Abstract

In animals, three main RNA interference mechanisms have been described so far, which respectively maturate three types of small noncoding RNAs (sncRNAs): miRNAs, piRNAs, and endo-siRNAs. The diversification of these mechanisms is deeply linked with the evolution of the Argonaute gene superfamily since each type of sncRNA is typically loaded by a specific Argonaute homolog. Moreover, other protein families play pivotal roles in the maturation of sncRNAs, like the DICER ribonuclease family, whose DICER1 and DICER2 paralogs maturate respectively miRNAs and endo-siRNAs. Within Metazoa, the distribution of these families has been only studied in major groups, and there are very few data for clades like Lophotrochozoa. Thus, we here inferred the evolutionary history of the animal Argonaute and DICER families including 43 lophotrochozoan species. Phylogenetic analyses along with newly sequenced sncRNA libraries suggested that in all Trochozoa, the proteins related to the endo-siRNA pathway have been lost, a part of them in some phyla (i.e. Nemertea, Bryozoa, Entoprocta), while all of them in all the others. On the contrary, early diverging phyla, Platyhelminthes and Syndermata, showed a complete endo-siRNA pathway. On the other hand, miRNAs were revealed the most conserved and ubiquitous mechanism of the metazoan RNA interference machinery, confirming their pivotal role in animal cell regulation.

**Significance**

In animals, three main RNA interference mechanisms have been described so far, called miRNAs, piRNAs, and endo-siRNAs, along with proteins involved in their maturation; among animals, the distribution of these pathways has been only studied in major groups and knowledge from many phyla is poor. We inferred the evolutionary history of the proteins maturating these RNAs, namely the animal Argonaute and DICER families, including 43 lophotrochozoan species. We describe the loss of the endo-siRNA pathway during the evolution of Lophotrochozoa: some phyla exhibit the complete pathway, while other phyla show a partial processing machinery or no machinery at all—contrastingly, miRNAs were revealed the most conserved and ubiquitous mechanism of the animal RNA interference machinery.

## Introduction

Argonaute proteins are cytoplasmic proteins that play a key role in most of the RNA interference (RNAi) pathways. They interact with a small noncoding RNA (sncRNA), forming an RNA-induced silencing complex (RISC). This ribonucleoprotein complex binds and silences target transcripts, using the complementary sncRNA as a probe (Iwakawa and Tomari 2022). Argonaute proteins can be found throughout most eukaryotic clades and share a common structure, featuring four domains: *N*-terminal (N), PIWI-Argonaute-Zwille (PAZ), Middle (MID), and P element-induced wimpy testis (PIWI) (Kuhn and Joshua-Tor 2013). The PIWI domain resembles the RNAse H domain's structure, but only some Argonautes have been reported to cleave the target mRNA (Song et al. 2004). All the other Argonaute proteins repress the target trough proteins that interact with the RISC complex (Huntzinger and Izaurralde 2011; Wu et al. 2020).

Among the Argonaute superfamily, four different families have been characterized: *Trypanosoma*-AGO family, WAGO family, AGO-like family, and PIWI-like family (Swarts et al. 2014). *Trypanosoma*-AGO proteins have been identified only in the euglenozoan order Trypanosomatida (Garcia Silva et al. 2010). Conversely, WAGOs have been characterized in nematodes only, whereas AGO-like and PIWI-like proteins seem to be found in all animal phyla (Höck and Meister 2008; Swarts et al. 2014).

Three classes of metazoan interfering sncRNAs can be identified: micro-RNAs (miRNAs), small interfering RNAs (siRNAs), and piwi-interacting RNAs (piRNAs) (Iwakawa and Tomari 2022). Each class is matured by different pathways, is loaded by a different Argonaute protein, and plays different cellular functions. Precursors of miRNAs (i.e. pri-miRNAs) are encoded by specific genes, transcribed by the RNA polymerase II (Lee, Kim, et al. 2004; Bartel 2018). Pri-miRNAs feature a hairpin secondary structure, with single-stranded ends at their 3′ and 5′ (Bartel 2018). These precursors undergo several maturation steps, starting immediately inside the nucleus, where they are targeted by the microprocessor complex, which cleaves the overhanging nucleotides of pri-miRNAs, leaving a stem-loop structure with a 2 bp offset, named pre-miRNA (Lee et al. 2003). Then, pre-miRNAs are exported in the cytoplasm, where they become substrate for DICER1, an endonuclease featuring one PAZ domain and two RNase III domains (Bernstein et al. 2001; Lee, Nakahara, et al. 2004). Through its catalytic activity, DICER1 removes the hairpin's loop, leaving a ∼22 bp ds-miRNA (Bernstein et al. 2001; Bartel 2018). Eventually, an AGO-like Argonaute binds to the ds-miRNA in the cytoplasm and disposes of one of the two strands, consequently resulting in a RISC complex. The miRNA-guided RISCs mostly target mRNAs by binding their 3′ untranslated region (UTR), interfering with their stability (Bartel 2018).

Unlike miRNAs, siRNAs may apparently be obtained from roughly every RNA capable of assuming a dsRNA structure (Shabalina and Koonin 2008). Therefore, siRNAs can originate from transcripts of transposons, repeated elements, or pseudogenes (Czech et al. 2008; Tam et al. 2008; Malone and Hannon 2009). siRNAs undergo a maturation pathway that is very similar to that of miRNAs, leading to the hypothesis that they evolved from a common ancestral RNAi system (Shabalina and Koonin 2008; Moran et al. 2017). Once the dsRNA precursors reach the cytoplasm, they are processed by DICER2, a paralog of DICER1. DICER2 cleaves a ∼21 bp dsRNA that is loaded into an AGO-like Argonaute, called AGO2 in fruit flies (Lee, Nakahara, et al. 2004; Matranga and Zamore 2007; Czech et al. 2008). The resulting siRISC complex maintains one of the two strands, again using it as a probe (Matranga and Zamore 2007).

In insects, the siRNA-mediated RNAi activity is not restricted to endogenous dsRNAs, but DICER2 is also able to target dsRNAs of viral origin, producing exogenous siRNAs that are pivotal for the innate immune response (Schuster et al. 2019). *Caenorhabditis elegans* expresses a single DICER paralog, which processes endogenous and viral dsRNAs, but also primary miRNA structures (Welker et al. 2011). Nematodes are even capable of producing secondary siRNAs thanks to the RNA-dependent RNA polymerases (Matranga and Zamore 2007), which are absent in mammals and insects. In mammals, viral dsRNAs are targeted by RIG-I-Like receptors (Loo and Gale 2011), which induce an antiviral response through the activation of type I interferons (Isaacs et al. 1963; Schuster et al. 2019). The ability to process long dsRNAs seems to be related to the DICER helicase domain, which is functional in *C. elegans* DICER and insects’ DICER2 (Welker et al. 2011; Sinha et al. 2018; Aderounmu et al. 2023). Contrastingly, the helicase function is not required for DICER proteins that are mainly involved in miRNAs maturation (i.e. insects’ DICER1 and mammals’ DICER; Jiang et al. 2005; Aderounmu et al. 2023).

Finally, piRNAs are 24 to 35-nt-long RNAs that originate from longer single-strand precursors, consisting of either active transposons or transcripts of genomic piRNA clusters (Hirakata and Siomi 2016). Once these ssRNA precursors are exported through the nuclear pores, they undergo a rather complex maturation pathway, which takes place mostly in the perinuclear nuage (Weick and Miska 2014; Hirakata and Siomi 2016). Although piRNAs appear to be Metazoa-restricted (Grimson et al. 2008), biogenesis pathways vary significantly between clades (Weick and Miska 2014). Mature piRNAs interact with PIWI-like Argonautes, resulting in a piRISC that operates as a defense system against transposons, by means of its nuclease activity (Malone and Hannon 2009). Moreover, piRISCs have been linked to specific epigenetic modifications of chromatin (i.e. H3K9me3) (Le Thomas et al. 2013). A peculiar piRNA amplification pathway called “ping-pong cycle” has been characterized in fruit flies and later discovered in mice as well (Brennecke et al. 2007; Weick and Miska 2014). This amplification pattern is performed by two different PIWI-like Argonautes, namely one that binds sense-piRNAs (AGO3 in fruit flies) and one that loads antisense piRNAs (Aubergine in fruit flies). As soon as one of these two proteins cleaves its target, it yields a secondary piRNA that can be loaded on the other PIWI-like protein, generating an amplification loop (Weick and Miska 2014; Hirakata and Siomi 2016). Another type of piRNA amplification is called “phasing.” PIWI loads a single-strand RNA (pre-pre-piRNA) from the 5' end, directing the endonucleolytic cleavage of the ribonuclease Zucchini at the 3′ end. This process is repeated along the pre-pre-piRNA leading to phased matured piRNAs (Mohn et al. 2015; Ozata et al. 2019).

miRNAs, piRNAs, and endo-siRNAs were likely to be in the last metazoan common ancestor, since they have been described in Porifera, Cnidaria, and most of the metazoan phyla (Grimson et al. 2008; Wheeler et al. 2009; Moran et al. 2013; Praher et al. 2017; Calcino et al. 2018; Fridrich et al. 2020). In some clades, piRNA and endo-siRNA pathways have been secondarily lost (Wynant et al. 2017; Fontenla et al. 2021), while the miRNA pathway is likely to be ubiquitous in animals (Fromm et al. 2022).

RNAi mechanisms are far less studied in Lophotrochozoa than in Deuterostomia and Ecdysozoa. This superclade includes around 14 phyla, which is notably higher than Ecdysoza and Deuterostomia, which comprises respectively eight and three phyla (Brusca et al. 2016). Moreover, Lophotrochozoa show an astonishing variability in body plans. For medical and nutritional reasons, most of the data are restricted to Mollusca and Platyhelminthes. Parasitic Platyhelminthes (i.e. Neodermata) lack the PIWI pathway, but the miRNA and endo-siRNA pathways have been reported (Fontenla et al. 2017; Fontenla et al. 2021). The PIWI pathway has been confirmed in Mollusca, where a clear signature of ping-pong amplification is visible (Jehn et al. 2018), and many miRNAs have been annotated in mollusks (Fromm et al. 2022). On the other hand, proteins related to the endo-siRNA pathway are absent in Bivalvia (Rosani et al. 2016). Outside those phyla, few data have been published. The endo-siRNA pathway seems absent in the Annelida *Capitella teleta* (Khanal et al. 2022) and the annotation of miRNA families is restricted to one Syndermata, one Brachiopoda, and two Annelida species (Fromm et al. 2022).

The phylogenetic relationships within Lophotrochozoa have been strongly debated, and they are not fully resolved yet. Morphological analyses agree to include Mollusca, Annelida, Brachiopoda, Phoronida, Nemertea, Bryozoa, Entoprocta, and Cycliophora in the Trochozoa clade (f.i., Kocot 2016). Some molecular analyses proved to be concordant with the Trochozoa clade (Struck et al. 2014; Laumer et al. 2015; Kocot et al. 2017; Laumer et al. 2019), but some others did not (Kocot et al. 2017; Marlétaz et al. 2019). Even the relationships among Trochozoa are far from being resolved, namely the monophyly of Polyzoa (Bryozoa + Entoprocta + Cycliophora) has regained credit recently (Khalturin et al. 2022; but see (Nesnidal et al. 2013; Kocot 2016; Bleidorn 2019; Laumer et al. 2019). Outside Trochozoa, Rouphozoa (Platyhelminthes + Gastrotricha) and Chaetognathifera (Syndermata + Micrognathozoa + Gnathostomulida + Chaetognatha) are supported by most of the phylogenetic analyses (Struck et al. 2014; Laumer et al. 2015; Kocot et al. 2017; Laumer et al. 2019; Conway Morris et al. 2020).

In recent years, the increase in -omics data has made it possible to compare and study the evolution of protein families along Lophotrochozoa. In this study, we exploited various -omics resources from nine lophotrochozoan phyla to annotate and characterize the diversification of the Argonaute and DICER proteins. We also analyzed sncRNA libraries to annotate the three sncRNA types and confirm the presence or absence of a particular sncRNA type in some phyla. According to our results, along the Lophotrochozoa evolution, the endo-siRNA pathway has been progressively lost, starting with DICER2 in Trochozoa, followed by the loss of the fruit fly AGO2-like proteins in Phoronida, Brachiopoda, Annelida, and Mollusca. This pattern is confirmed by the distribution of DICER2 and AGO2-like proteins in the analyzed organisms. In contrast, the piRNA and miRNA pathways appeared to be conserved in almost all Lophotrochozoa.

## Results

### The Argonaute and DICER Phylogeny

We annotated Argonaute proteins of 43 lophotrochozoan species by analyzing 19 proteomes, 16 genomes, and 8 transcriptomes. Argonaute sequences of *Homo sapiens* (Chordata), *Drosophila melanogaster* (Arthropoda), *C. elegans* (Nematoda), and *Nematostella vectensis* (Cnidaria) were retrieved from SwissProt and included in the phylogenetic analysis as references (supplementary table S1, Supplementary Material online). Moreover, we included metazoan species whose RNAi pathways have already been studied (see Introduction), testing whether our Argonaute and DICER annotation matches results from the literature (Table 1).

**Table 1:**
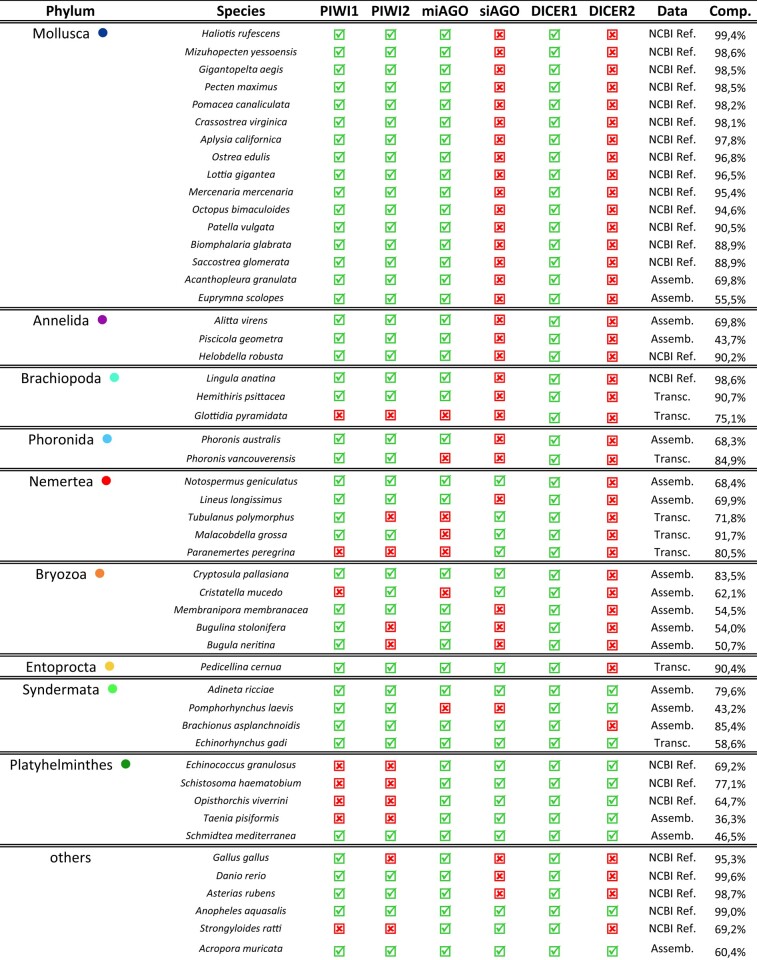
Presence and absence of Argonaute and DICER proteins.

**Fig. 1.**
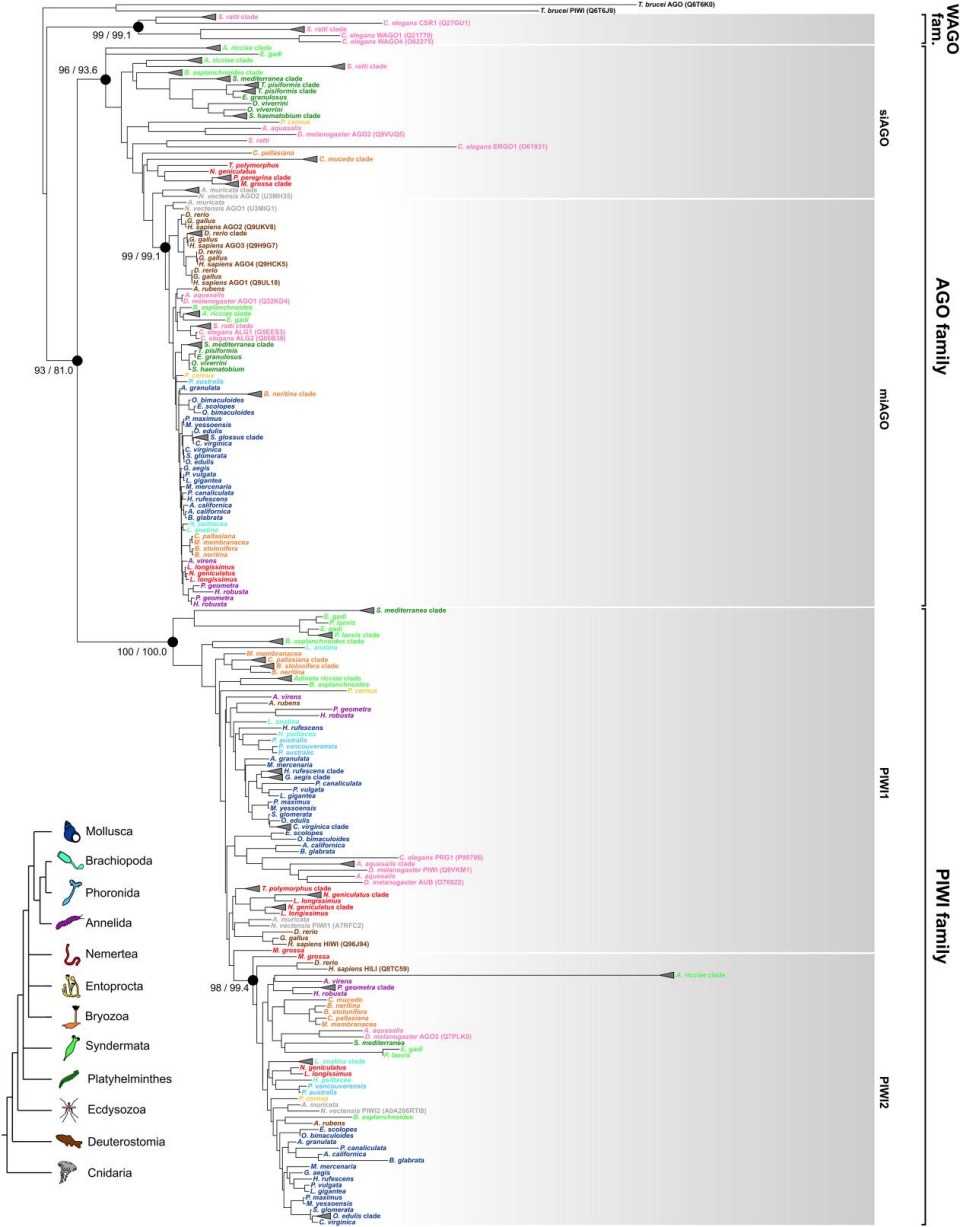
ML tree of the lophotrochozoan Argonaute proteins. For the six marked nodes, the label shows UFBoot/SH-alrt value. Support values of the remaining nodes are shown in supplementary fig. S1, Supplementary Material online. Clades formed by paralogs of the same species were collapsed and represented with a triangle. For reference proteins, the UniProt accession code is reported in brackets. Species are colored according to the phylum. The color legend on the bottom left reconstructs the main phylogenetic relationships according to the latest Lophotrochozoa phylogenetic analyses (Kocot et al. 2017; Bleidorn 2019; Marlétaz et al. 2019).

**Fig. 2.**
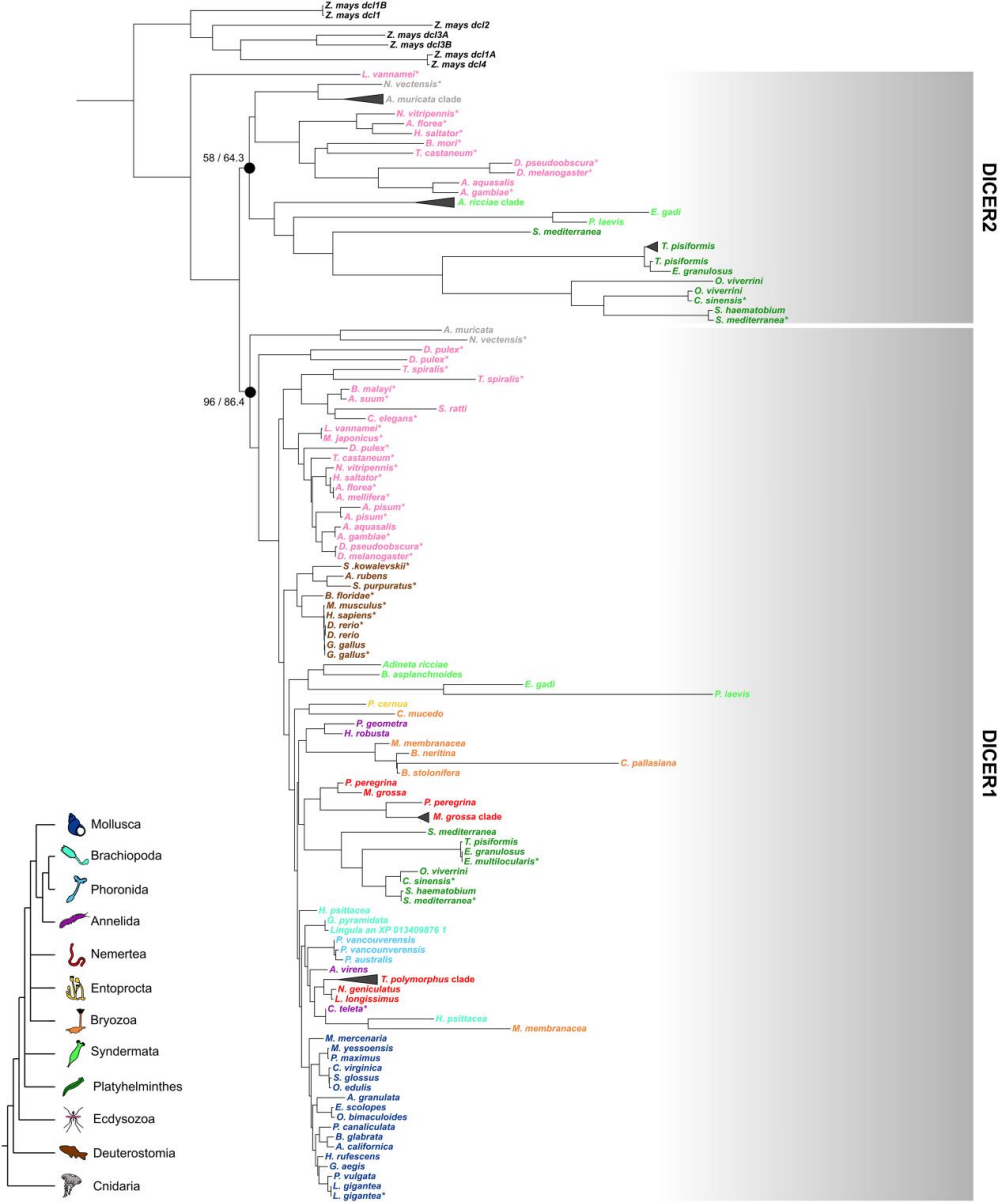
ML tree of the lophotrochozoan DICER proteins. For two marked nodes, it is reported respectively the UFBoot and the SH-alrt value. Support values of the remaining nodes are shown in the supplementary fig. S2, Supplementary Material online. Clades formed by paralogs of the same species were collapsed and represented with a triangle. Reference species retrieved from the analysis of Mukherjee and colleagues (2013) are marked with an asterisk. Species are colored according to the phylum. The color legend on the bottom left reconstructs the main phylogenetic relationships according to the latest Lophotrochozoa phylogenetic analyses (Kocot et al. 2017; Bleidorn 2019; Marlétaz et al. 2019).

Colored dots refer to colors assigned to the different animal phyla in Figs. 1 and 2. For the four Argonaute proteins and the two DICER proteins, a green check marks the species where the protein has been annotated, and a red cross marks species where the protein is absent. The “Data” column reports the type of data, proteome annotated following the NCBI Eukaryotic Genome Annotation Pipeline (“NCBI Ref.”), a genome assembly (“Assemb.”), or a transcriptome (“Transc.”). The last column reports the BUSCO completeness score.

All the annotated Argonaute proteins were aligned, and the maximum-likelihood (ML) tree was inferred. The PIWI and AGO proteins of *Trypanosoma brucei* were obtained from UniProt and used as outgroups (supplementary table S1, Supplementary Material online). The phylogenetic tree of the Argonaute superfamily supported the known main families (Fig. 1; see supplementary fig. S1, Supplementary Material online, for the uncollapsed tree with support values for each node). The WAGO family, which is restricted to nematodes, included WAGOs and CSR-1 sequences from *C. elegans*. The PIWI-like family was characterized by AGO3, PIWI, and AUB of *D. melanogaster*, HILI and HIWI of *H. sapiens*, and PRG-1 of *C. elegans*; the AGO-like family comprised AGO1,2 of *D. melanogaster*, AGO1 to 4 of *H. sapiens*, and ALG1,2 of *C. elegans*. Every family was widely supported by ultrafast bootstrap approximation (UFBoot) and the SH-like approximate likelihood ratio test (SH-alrt test) (Fig. 1).

Within each family, it was possible to identify the different Argonaute proteins. Argonaute proteins related to miRNAs were characterized by proteins like *H. sapiens* AGO1 to 4, *D. melanogaster* and *N. vectensis* AGO1, and *C. elegans* ALG1,2 and resulted in a monophyletic clade (UFBoot = 99 and SH-alrt = 99.1; Fig. 1). Almost all lophotrochozoan species featured a protein clustering within this clade, with very few exceptions, and at least one organism from each phylum was recovered in this clade (Fig. 1; Table 1). We will refer to this clade as the “miAGO clade.” The remaining proteins within the AGO-like family were recovered as paraphyletic with respect to the miAGO clade. This group included *D. melanogaster* AGO2, *N. vectensis* AGO2, and *C. elegans* ERGO, all proteins that target endo-siRNAs (but with some exceptions: see Fridrich et al. 2020). Actually, endo-siRNA proteins are often inferred as paraphyletic with respect to the miAGO clade (Swarts et al. 2014; Praher et al. 2017; Wynant et al. 2017); we will call this group the “siAGO group.” Few lophotrochozoan phyla are included in this group, since we annotated at least one siAGO protein for all the Platyhelminthes and Entoprocta species, while three other phyla featured at least one species within the group, namely Nemertea, Bryozoa, and Syndermata. We did not retrieve any siAGO protein from the remaining clades, namely Mollusca, Annelida, Brachiopoda, and Phoronida (Fig. 1; Table 1).

PIWI-like proteins showed a pattern similar to that of the AGO-like family. PIWI2 proteins were clustered in a monophyletic clade, characterized by already annotated PIWI2 proteins, like *H. sapiens* HILI, *N. vectensis* PIWI2, and *D. melanogaster* AGO3; all the remaining PIWI-like proteins are paraphyletic with respect to PIWI2 proteins. This grade included already annotated PIWI1 proteins like *H. sapiens* HIWI, *N. vectensis* PIWI1, *C. elegans* PRG1, and *D. melanogaster* AUB and PIWI. Both PIWI-like protein groups included at least one protein from each lophotrochozoan phylum (Table 1). PIWI-like proteins were almost absent in Platyhelminthes, apart from *Schmitdea mediterranea* (Fig. 1).

Regarding the six nonlophotrochozoan species included in the analysis, the annotation of Argonaute proteins matched the expectations: miAGO proteins were retrieved for all six species; siAGO proteins were absent in *Danio rerio*, *Gallus gallus*, and *Asterias rubens* (Deuterostomia); in all six species, we annotated PIWI1 and PIWI2 proteins, exception made for *Strongyloides ratti* (i.e. that lacked of both PIWI proteins) and, unexpectedly, *G. gallus* that lacked of PIWI2 (Fig. 1; Table 1).

The phylogenetic analysis highlighted the presence of miAGO proteins in each phylum, but only some of them featured siAGO proteins. In Arthropoda, the precursor structures of siRNAs and miRNAs are processed by two distinct DICER paralogs: DICER2 and DICER1, respectively (Shabalina and Koonin 2008). DICER2 has been found in other phyla, such as Cnidaria and Platyhelminthes (Mukherjee et al. 2013), while clades lacking siAGO proteins lack DICER2 as well. We annotated DICER proteins and inferred the phylogeny to understand whether Lophotrochozoa follow the same pattern.

We annotated DICER proteins querying the lophotrochozoan sequences against an annotated metazoan DICER set (Mukherjee et al. 2013) and looking for the ribonucleases 3 domain. The phylogenetic analysis included the metazoan DICER set of Mukherjee and colleagues (2013) as references, including the *Zea mays* DICER proteins as outgroups (Fig. 2; see supplementary fig. S2, Supplementary Material online, for the uncollapsed tree with support values for each node). The resulting ML tree clustered the DICER proteins into two distinct groups. Recall the position of the reference sequences, DICER1 and DICER2 sequences, are accordingly split into the two groups, apart from *Litopenaeus vannamei* DICER2, which is basal to all the other proteins. The monophyly of the DICER1 group is supported by the SH-alrt test (86.4) and the UFBoot (96). In contrast, the low support values of the DICER2 node (UFBoot = 58 and SH-alrt = 64.3) undermine the hypothesis of a unique common origin of DICER2 proteins. Nonetheless, the presence of DICER2 was restricted to a few lophotrochozoan phyla: all Platyhelminthes and three Syndermata species showed a DICER2 protein. On the other hand, DICER1 was annotated for every phylum: thus, Mollusca, Annelida, Phoronida, Brachiopoda, Nemertea, Bryozoa, and Entoprocta reported DICER1 proteins, but no DICER2 proteins (Fig. 2; Table 1). In line with expectations, DICER2 was found in *Acropora muricata* and *Anopheles gambiae*, while it was lacking in *D. rerio*, *G. gallus*, *A. rubens*, and *S. ratti*. In contrast, DICER1 was annotated in all of them.

Overall, the DICER family phylogenetic analysis confirmed that the absence of siAGO proteins coincides with the absence of DICER2, and vice versa, exception made for Nemertea, Bryozoa, and Entoprocta, where we annotated siAGO proteins for most species; however, none of these species featured DICER2. DICER1 and miAGO proteins showed lower evolutionary rates than their paralogous counterparts: the root-to-tip distances of miAGO branches were significantly lower than the root-to-tip distances of siAGO, PIWI1, and PIWI2 branches, and the root-to-tip distances of DICER1 branches were significantly lower than their DICER2 counterparts (supplementary fig. S3, Supplementary Material online). We also estimated the ratio of nonsynonymous to synonymous substitution rates (*ω*) along the AGO family tree and the DICER family tree: we confirmed that, during the evolution of the DICER and AGO families, purifying selection on DICER1 (LRT = 34.34, *P*-value = 4.62 × 10^−9^; supplementary fig. S4B, Supplementary Material online) and miAGO (LRT = 204,64, *P*-value = 0; supplementary fig. S4A, Supplementary Material online) has intensified compared with the rest of the family tree (Wertheim et al. 2015).

In both phylogenetic analyses, the presence of a protein in some phyla was not confirmed by all the species. To understand whether it might be related to the quality of the data, we evaluated the completeness of proteomes, genomes, and transcriptomes: Argonaute and DICER proteins were missing more commonly in transcriptomes than in proteomes or genome assemblies, regardless of completeness (Table 1). All proteomes showed a similar presence/absence pattern and high completeness values, while the completeness of the genomes varied significantly between species. In general, in more complete genome assemblies, we were also able to annotate more proteins (Table 1). Overall, phyla that are generally more represented in protein databases (i.e. Mollusca, Annelida, Platyhelminthes) showed a more constant presence/absence pattern between species than underrepresented phyla (i.e. Nemertea, Bryozoa, Syndermata). The annotation of genome assemblies with BRAKER has heavily relied on protein databases (see Materials and Methods); thus, it may be possible that this method produced better annotations for clades like Mollusca, Annelida, or Platyhelminthes.

### Domain Characterization in Argonaute and DICER Proteins

The annotation of Argonaute and DICER proteins mostly relied on the annotation of peculiar domains (see Materials and Methods). However, other domains characterize the two protein families. Thus, we built domain profiles of Argonaute and DICER domains from multiple sequences alignments. Profiles were aligned against the annotated proteins to evaluate the domain composition of lophotrochozoan Argonaute and DICER proteins.

Besides the PIWI and PAZ domains, Argonaute proteins are also characterized by N-terminal (N) and the MID domain. We failed to annotate the two domains in most of PIWI proteins; in particular, the MID domain was not annotated in any Syndermata and Bryozoa PIWI1 protein, but also in each lophotrochozoan phylum, we reported at least one species without the domain (supplementary table S2, Supplementary Material online). Additionally, each Syndermata PIWI2 protein lacked not only the MID domain but also the N domain. Similarly, Bryozoa PIWI2 proteins did not contain the MID domain either (supplementary tables S2 and S3, Supplementary Material online). On the other hand, in almost all miAGO proteins both domains were annotated (*Hemithiris psittacea* being the only exception), while in siAGO proteins, almost all Platyhelminthes lacked the MID domain. After localizing the domain position in the Argonaute alignment, we examined whether certain species lacked the domains entirely. This was determined by assessing whether their sequence in that portion of the alignment was either entirely absent (i.e. with most sites being gaps) or significantly degenerated (with most sites containing amino acids, but the sequence being too degenerated for accurate domain annotation). In most cases, the sequences were found to be degenerated (supplementary table S4, Supplementary Material online).

Regarding DICER domain composition, we assessed the presence of the Helicase (Hel) and PAZ domain. The Hel domain was absent in most of Lophotrochozoa DICER2 proteins, with the only exception of two Syndermata paralogs (supplementary table S5, Supplementary Material online). The PAZ domain also resulted absent from most of Lophotrochozoa DICER2 proteins, exception made for one *S. mediterranea* DICER2 paralog (supplementary table S6, Supplementary Material online). Among DICER1 proteins, Hel and PAZ domains were annotated in all Mollusca species, while in other lophotrochozoan phyla, the annotation of both domains was restricted to some species (supplementary tables S5 and S6, Supplementary Material online). Notably, the two domains resulted completely absent in Bryozoa. Contrastingly with the Argonaute analysis, DICER domains, when not recovered, were completely missing. According to the structure of DICER, the PAZ and Hel domains are toward the N-terminal end, with the Hel being the first domain of the protein (Mukherjee et al. 2013). Accordingly, all the lophotrochozoan DICER proteins lacking either the two domains or solely the Hel domain were found to be truncated at the N-terminus. The only exceptions were some DICER2 paralogs of the syndermatan *Adineta ricciae*: while five paralogs resulted truncated, three of them displayed degeneration only (supplementary table S4, Supplementary Material online).

We also evaluated the conservation of the DECH box within the Hel domain. The amino acid composition of the DECH box (i.e. aspartic acid, glutamic acid, cysteine, and histidine) resulted conserved in Syndermata, Nematoda, Phoronida, and Brachiopoda. Most Annelida and Gastropoda showed an aspartic acid instead of a glutamic acid on the second position (resulting in DDCH); the DECH box resulted further mutated in Bivalvia (i.e. ENCH in Ostreida, DHCQ in Pectinida, DDCH in *Mercenaria mercenaria*), in *Biomphalaria glabrata* (DNCH), and in Cephalopoda (ECSN). Platyhelminthes also reported a highly diverged DECH box (supplementary fig. S5, Supplementary Material online).

### Looking for the Endo-siRNA Signature in Small RNA Libraries

**Fig. 3.**
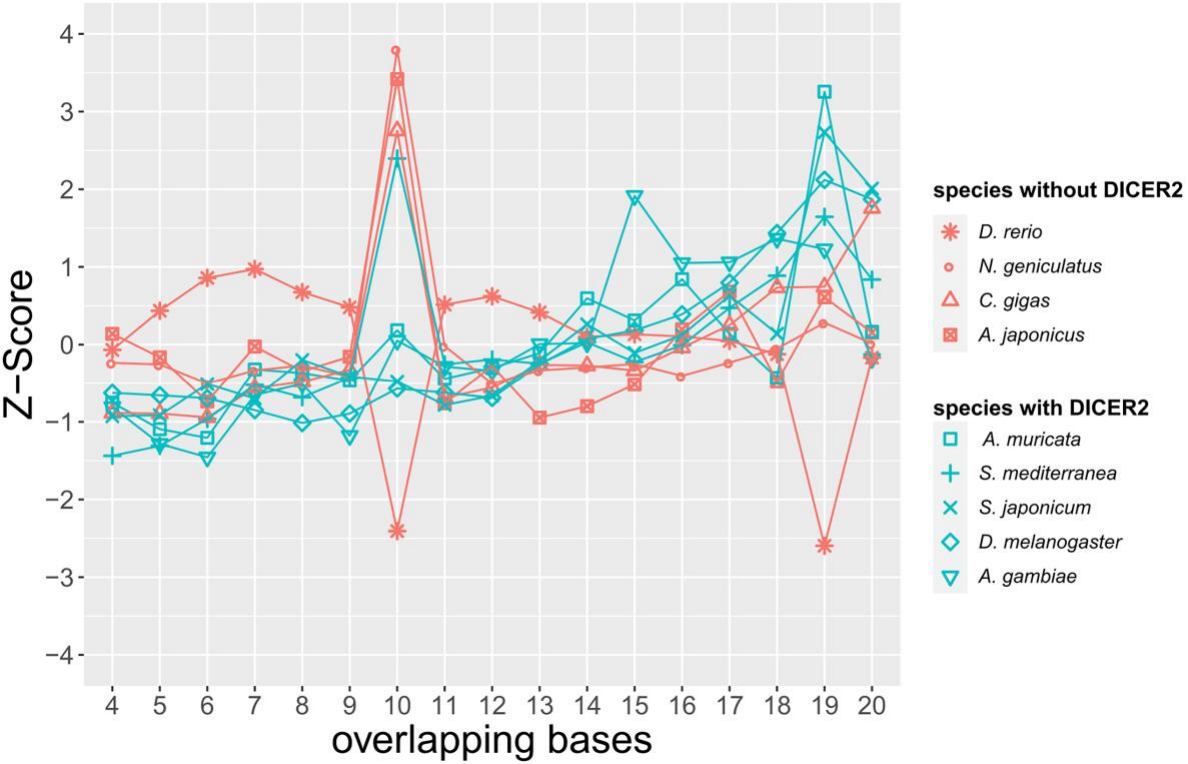
Evaluating the siRNA signature in sncRNA libraries. The plot reports the *Z*-score between the number of pairs of all possible overlaps. A *Z*-score > 1 means that pairs overlapping of that length are at least a standard deviation more numerous than the mean of all the overlaps. The species are sorted by the presence/absence of DICER2.

According to the phylogenomic analysis, some lophotrochozoan phyla lack pivotal proteins related to the endo-siRNA pathway. DICER2 generally processes double-stranded RNAs producing two 21 bases siRNA duplexes that overlap by 19 bases. Similarly, piRNAs produced by the ping-pong cycle go in pairs that overlap by 10 bases (Antoniewski 2014; Khanal et al. 2022). Thus, siRNAs and piRNAs have a unique signature that can be identified in sncRNA libraries by looking for overlapping pairs of reads. We retrieved eight sncRNA libraries from lophotrochozoan and nonlophotrochozoan species (namely *D. rerio*, *Apostichopus japonicus*, *A. muricata*, *A. gambiae*, *D. melanogaster*, *S. mediterranea*, *Schistosoma japonicum*, *Crassostrea gigas*), and we sequenced the sncRNA pool of *Notospermus geniculatus* (Nemertea) to include a species with an incomplete endo-siRNA pathway (i.e. presence of siAGOs, but absence of DICER2) in our analysis. Using the overlapping_reads.py script (Antoniewski 2014), we calculated the number of read pairs that overlapped for the same number of bases, from 4 to 20 bases. Then, we calculated the *Z*-score among the number of read pairs for each overlap group. A *Z*-score equal to 1 means that the number of read pairs in that overlap group is one standard deviation higher from the mean size of all the overlap groups of a given species. Taking into consideration only 21 bp reads (i.e. the expected length of endo-siRNAs), species equipped with DICER2 (i.e. *D. melanogaster*, *A. gambiae*, *A. muricata*, *S. mediterranea*, and *S. japonicum*) reported a *Z*-score higher in the 19-overlap group than species without DICER2 (*D. rerio*, *A. japonicus*, *C. gigas*, *N. geniculatus*; Fig. 3). Some species also reported a sharp increase in the *Z*-score for the 10-overlap group for 21 bp long reads only, namely *N. geniculatus*, *A. japonicus*, *C. gigas*, and *S. mediterranea*. A 10-base overlap would correspond to the piRNA signature, although the length of piRNAs in Arthropoda ranges from 25 to 30 bp. Overall, considering the phylogenomic analysis, all the species that exhibited a complete siRNA pathway (using DICER2 and siAGO as a proxy) showed a high *Z*-score in the 19-overlap group (Fig. 3). Finally, we investigated the range of action of the three sncRNA types in three metazoan species (i.e. *C. gigas* for Lophotrochozoa, *A. gambiae* for Ecdysozoa, and *D. rerio* for Deuterostomia). We annotated miRNAs, piRNAs, and endo-siRNAs evaluating their expression at different sncRNA lengths (supplementary fig. S6A, Supplementary Material online). In terms of reads per million (RPM), in all three species, miRNAs are the most expressed and their length ranges from 20 to 25 nc. In contrast, piRNAs were annotated in the in the 24 to 30 nc length range. In most cases, we were not able to discern the endo-siRNA signal from noise, but *A. gambiae* was the species that showed the highest expression levels (supplementary fig. S6A, Supplementary Material online). We also compared ovary and somatic tissue sncRNA libraries. As expected, in all three species, piRNAs is the class more expressed in the ovaries (supplementary fig. S6B, Supplementary Material online). Overall, in the three species, we did not report notable differences in terms of sncRNA length or differential expression in somatic/ovarian tissue among piRNA and miRNA types.

The annotation of the sRNA types was performed even for the newly sequenced small RNA libraries of *N. geniculatus*. Following the guidelines of Fromm et al. (2015), we identified 154 bona fide miRNA genes. Each miRNA gene has been annotated by blasting its preliminary structure against the MirGeneDB pre-miRNA database (Fromm et al. 2022) and checking the conservation of the seed region. According to the annotation results, 63 *N. geniculatus* miRNA genes were included in 29 miRNA families already described in other metazoan species (supplementary table S7, Supplementary Material online). The remaining 91 miRNA genes did not exhibit significant similarities with the pre-miRNAs in the database (supplementary materials, Supplementary Material online). To assess the conservation of the novel miRNAs and increase the reliability of our predictions, we aligned the pre-miRNAs against the *Lineus longissimus* genome (i.e. the most closely related species to *N. geniculatus* with an assembled genome). We identified at least 28 novel miRNA genes that are shared between *N. geniculatus* and *L. longissimus*. Based on their preliminary sequences and seed regions, we clustered these 28 novel miRNA genes into 17 novel families (supplementary table S8, Supplementary Material online). Comparing the expression levels of the three different types of small RNAs, considering small RNAs with a length ranging between 20 and 24 nucleotides, miRNAs resulted the most expressed even in *N. geniculatus*. Conversely, piRNAs resulted expressed mostly in the length range 25 to 29 nc (supplementary fig. S7, Supplementary Material online). As for *C. gigas* (supplementary fig. S6B, Supplementary Material online), we were not able to discern the endo-siRNA signal from noise.

## Discussion

### Most Lophotrochozoans Have Conventional miRNA and piRNA Pathways

RNAi pathways play a central role in many molecular aspects, from mRNA regulation to defense mechanisms, and Argonaute proteins are the key RNAi players in all eukaryotes. All the phylogenetic analyses agree to divide the eukaryotic Argonaute superfamily into four main families, namely the *Trypanosoma*-AGO family, the WAGO family, the AGO family, and the PIWI family (Höck and Meister 2008; Garcia Silva et al. 2010; Swarts et al. 2014). Excluding the *Trypanosoma*-AGO family, all the other families are represented within animals; moreover, the PIWI and the WAGO families are restricted to animals or even nematodes, respectively (Swarts et al. 2014). It is still uncertain how the four families emerged during eukaryote evolution. For instance, several eukaryotic clades have a miRNA-like pathway, but it is not clear whether these pathways are analogous or homologous to the metazoan miRNA pathway (Moran et al. 2017). It is likely that RNAi systems diverged from an ancestral siRNA system and, considering the distribution of Eukaryota clades in the four families (Swarts et al. 2014), the divergence took place at least 1.5 billions of years ago (Strassert et al. 2021).

The inferred Argonaute phylogenetic tree confirmed and highly supported the three metazoan Argonaute families (Fig. 1). Moreover, we identified two distinct groups in the AGO and PIWI, where only one of the two groups is monophyletic. This pattern is confirmed in other Argonaute phylogenetic analyses (Swarts et al. 2014; Praher et al. 2017; Wynant et al. 2017). Recalling the deep divergence of these proteins, the signal might be saturated. Accordingly, most of the nodes at the base of the family are not strongly supported (Table 1). The same pattern has been observed in the DICER phylogeny, with DICER2 proteins being paraphyletic with respect to DICER1 proteins, which clustered in a well-supported monophyletic clade (Fig. 2) (Mukherjee et al. 2013). Concordantly, DICER2 and DICER1 are related to siAGO and miAGO proteins, respectively. Overall, within each clade, the phylogenetic reconstruction is substantially in agreement with the state-of-art animal phylogeny, recalling that the signal has been inferred from single markers.

Almost all lophotrochozoans showed two distant related PIWI proteins (i.e. one AUB-like and one AGO3-like; Fig. 1; Table 1), with the only exception of Neodermata (Platyhelminthes), which lacks the whole piRNA pathway (Fontenla et al. 2021). It is likely that the ping-pong cycle, which has already been described in some mollusks (Jehn et al. 2018), has been maintained in most of lophotrochozoan. The piRNA expression of *C. gigas* is in line with that of *D. rerio* and *A. gambiae*, and the differential expression analysis confirms that piRNAs are more expressed in the gonads in all three clades.

The miRNA pathway is the most ubiquitous RNAi pathway among Metazoa, and almost all species reported a miAGO and a DICER1 protein. For these proteins, we even detected lower root-to-tip distances and a decrease in *ω* along their branches, which confirms a higher selective pressure. Therefore, the high conservation of proteins involved in the miRNA pathway reflects the well-known conservation of miRNAs among Metazoa (Tarver et al. 2013).

Even in our case, we confirmed that the annotated miRNAs showed similar features in the three reference species; they are 20 to 25 nt long, they are not more expressed in the ovaries than in somatic tissue, and overall, they are by far the most expressed sncRNA class.

The conservation is also reflected in the domain composition; most of miAGO and DICER1 proteins included all the domains. However, a novel pattern has been observed in the DECH box. The DECH box is a motif present in many helicase domains, and it coordinates ATP hydrolysis (Yerukhimovich et al. 2018). Although it is conserved between distant related DICER proteins (f.i., *H. sapiens* DICER1, *D. melanogaster* DICER2, *C. elegans* DICER; supplementary fig. S5, Supplementary Material online), its conservation does not imply that the Hel domain is active, since the *H. sapiens* DICER has been proved to work in an ATP-independent manner (Liu et al. 2018). The Hel domain is likely inactive also in Mollusca and Annelida, where the DECH box diverges of one or two amino acids, but it remains conserved in Phoronida, Brachiopoda, and Nematoda, where it may be still active (supplementary fig. S5, Supplementary Material online).

### The Evolution of the Endo-siRNA Pathway in Lophotrochozoa

Pathways maturating endo-siRNAs are deeply diverse between metazoan clades. In the fruit fly, the miRNA and the endo-siRNA pathways are separated, having a specific Argonaute and DICER protein for each pathway. In *C. elegans*, a single DICER protein is responsible for the maturation of miRNAs and endo-siRNAs: endo-siRNAs are then loaded by ERGO-1 (but also other Argonaute proteins; Han et al. 2009). RNA-dependent RNA polymerases amplify the mechanism through the production of secondary siRNAs, loaded by WAGO proteins (Billi et al. 2014). In mammals, even if they lack siAGO proteins, endo-siRNAs are loaded on AGO2 (Watanabe et al. 2008), but their maturation has not been elucidated yet. Endo-siRNAs have been also reported in early diverging animals, where they are loaded by a specific siAGO (Fridrich et al. 2020).

Overall, small RNAs produced by long endogenous dsRNAs have been described in all Metazoa, but the maturation pathway evolved differently in different clades. This pathway has been certainly overlooked in Lophotrochozoa. An endo-siRNA pathway is likely to exist in most of early diverging Lophotrochozoa, since most of Platyhelminthes and Syndermata included a siAGO and DICER2 protein (Figs. 1 and 2). This endo-siRNA pathway looks like the one of insects and cnidarians, where two distinct DICER and AGOs interacts with two distinct small RNA types (at least for most small RNAs, see Fridrich et al. 2020). However, lophotrochozoan DICER2 proteins showed notable differences in the domain composition compared with cnidarian or insect ones. Most of them lack the Hel and PAZ domain, which are pivotal for DICER2. The PAZ domain recognizes target dsRNAs and binds their 3′ end, while the Hel domain, which seems to be inactive in all metazoan DICER1 (Aderounmu et al. 2023), allows the translocation of DICER2 along the target dsRNA, producing siRNAs processively (Kandasamy and Fukunaga 2016).

Nevertheless, we detected small RNAs with the peculiar endo-siRNA signature (i.e. 21 bp small RNA pairs with an overlap of 19 bases; Fig. 3) in the small RNA transcriptomes of *S. mediterranea* and *S. japonicum* (Platyhelminthes). Thus, it is possible that the DICER2 of early diverging Lophotrochozoa can still maturate dsRNAs without the PAZ and the Hel domain. When the Hel domain is experimentally inactivated in DICER2 of *C. elegans* or in *D. melanogaster*, the protein loses the ability of translocase along dsRNAs, but it is still able to target dsRNAs and produce endo-siRNAs (Welker et al. 2011; Sinha et al. 2018). At the same time, the inactivation of the PAZ domain leads to the production of siRNAs of altered length (Kandasamy and Fukunaga 2016). Overall, Platyhelminthes and Syndermata DICER2 might still work, and our results show that (Fig. 3), but the lack of the two domains might affect them in fidelity and efficiency.

**Fig. 4.**
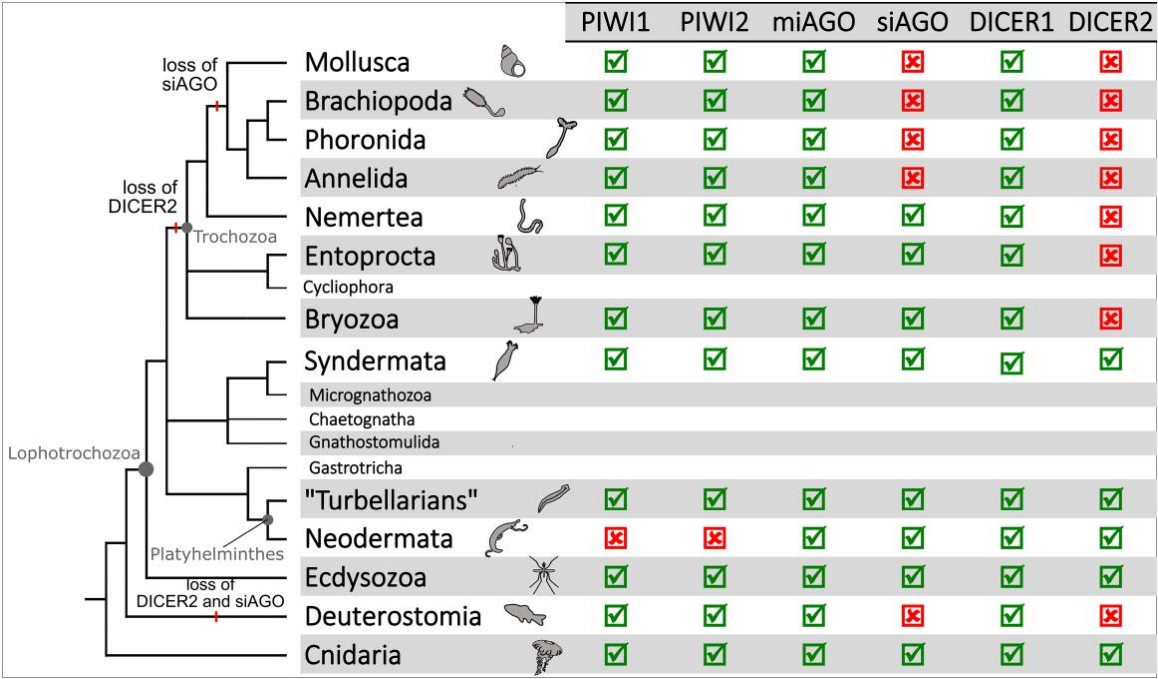
The loss of DICER2 and siAGO along the Metazoa evolution. The lophotrochozoan phylogenetic tree is reconstructed according to the latest Lophotrochozoa phylogenetic analyses (Kocot et al. 2017; Bleidorn 2019; Marlétaz et al. 2019). For each protein, it is reported its presence, with a check, or the absence, with a cross, in each metazoan clade.

Within Trochozoa, we did not detect DICER2, but some species belonging to Nemertea, Bryozoa, and Entoprocta possess siAGO proteins. These three phyla have occasionally been placed as sister group of all the other Trochozoa (Kocot 2016; Kocot et al. 2017; Laumer et al. 2019; Khalturin et al. 2022). Thus, the endo-siRNA pathway would have been progressively lost during the evolution of Lophotrochozoa. The first step has been the loss of DICER2 in the ancestor of Trochozoa. Then, the loss of siAGO proteins in the ancestor of Mollusca, Brachiopoda, Phoronida, and Annelida followed (Fig. 4). On the other hand, many other analyses do not place Entoprocta, Ectoprocta, and Nemertea at the base of Trochozoa (Laumer et al. 2015; Marlétaz et al. 2019); in that scenario, siAGO proteins would have been lost multiple times, depending on the phylogenetic relationships between phyla. Finally, the absence of a complete endo-siRNA pathway in Trochozoa is confirmed by the analysis of small RNA transcriptomes: we did not detect small RNAs with the endo-siRNA signature in species with an uncomplete endo-siRNA pathway (Fig. 3), namely *C. gigas* (Mollusca) and *N. geniculatus* (Nemertea), but also *D. rerio* and *A. japonicus* (Deuterostomia).

### Unraveling the Evolution of RNAi Pathways in Lophotrochozoa

In this study, we highlighted notable differences between Lophotrochozoa and other Metazoa RNAi pathways. Platyhelminthes and Syndermata have maintained an endo-siRNA machinery, but DICER2 diverges considerably from the DICER2 protein of other metazoan clades. Nevertheless, Platyhelminthes are able to produce endo-siRNAs (Fig. 3). Since the endo-siRNA pathway is highly diverse among different Metazoa clades, it is likely that also in early diverging Lophotrochozoa, the endo-siRNA pathway has evolved in a unique way, not completely comparable with other metazoan pathways.

An even more unique condition was described in Nemertea, Entoprocta, and Ectoprocta. These clades show an intermediate state during the loss of the endo-siRNA pathway in Lophotrochozoa. The absence of DICER2 proteins may preclude a functional endo-siRNA pathway. However, this pathway proved to be very flexible. It is possible that the siAGO protein of these clades has evolved to load small RNAs maturated from other pathways. Like in *C. elegans*, DICER1 may maturate miRNA as well as endo-siRNA. Having said that, there are multiple scenarios where siAGO proteins may be involved, also considering the number of unconventional RNAi pathways that have been described so far (Yang and Lai 2011).

Finally, the loss of siAGO proteins and, more generally, of the endo-siRNA pathway, possibly in all Trochozoa, has been strongly supported by the joint phylogenetic analysis and analysis of sncRNA libraries. The pattern is comparable with that obtained from Deuterostomia, where the absence of the canonical (i.e. involving both DICER and siAGOs) endo-siRNA pathway has already been reported using the lack of annotated siAGO proteins as a proxy (Wynant et al. 2017). Our analysis supports this hypothesis, since neither siAGO nor DICER2 homologs were retrieved in deuterostomes (Figs. 1 and 2). Nevertheless, in mammals, endo-siRNAs are processed by the same DICER and AGO proteins that process miRNAs (Watanabe et al. 2008; Svobodova et al. 2016). Similarly, in Trochozoa, endo-siRNAs may be matured by the miRNA pathway or other proteins related to the RNAi mechanism. As for mammals, immunoprecipitation or knockout experiments might elucidate whether Argonaute proteins, as well as other protein families, can interact with other sncRNAs types in addition to piRNAs and miRNAs. All these findings are also deeply linked to the characterization of the innate immune system. In mammals, the interferon pathway has replaced RNAi mechanisms in the role of viral defense (Isaacs et al. 1963; Loo and Gale 2011; Schuster et al. 2019). An interferon defense mechanism has been described also in Mollusca (Huang et al. 2017; Qiao et al. 2021). Thus, in Lophotrochozoa, an interferon system might have evolved coincidentally with the loss of the endo-siRNA pathway. Further comparative analyses might characterize the evolution of these pathways and elucidate the mechanisms that control the innate immune system in Lophotrochozoa.

## Materials and Methods

### Annotation of Argonaute and DICER Proteins

Argonaute and DICER proteins were annotated for a wide range of omics data. Initially, we analyzed all lophotrochozoan proteomes annotated through the National Center for Biotechnology Information (NCBI) Eukaryotic Genome Annotation Pipeline (Thibaud-Nissen et al. 2016). To increase the sampling in underrepresented clades, we selected 17 assemblies (supplementary table S9, Supplementary Material online) and predicted gene models using the BRAKER2 automated pipeline (Stanke et al. 2008; Hoff et al. 2019; Brůna et al. 2021). To enhance the DICER and Argonaute model predictions, we enriched the Metazoa OrthoDB database provided by BRAKER2 with Argonaute and DICER sequences annotated from the lophotrochozoan NCBI proteomes. We collapsed all isoforms, retaining the longest ones, using the Perl script agat_sp_keep_longest_isoform.pl (Dainat et al. 2019). When few genome assemblies were available for a given phylum, we searched for Argonaute and DICER proteins in transcriptomes (Table 1). We trimmed the reads with Trimmomatic-0.39 (Bolger et al. 2014) using the following settings: ILLUMINACLIP: TruSeq3-PE.fa:2:30:10 LEADING:3 TRAILING:3 SLIDINGWINDOW:4:15 MINLEN:75. We assembled the transcriptome with Trinity v2.1.1 with default settings (Grabherr et al. 2011); we filtered out contaminants by locally aligning the transcripts against the nonredundant protein database (Sayers et al. 2022) with DIAMOND blastp (Camacho et al. 2009; Buchfink et al. 2015) and discarding all transcripts with a nonmetazoan best hit.

Coding regions were predicted with TransDecoder v5.5.0 (https://github.com/TransDecoder/TransDecoder), scanning all ORFs for homology using DIAMOND blastp and HMMER (Mistry et al. 2013). Overall, we obtained the coding sequences of 42 lophotrochozoan species and 6 other metazoan species. Coding sequences were translated into amino acid sequences, and then, Argonaute and DICER proteins were annotated as follows: we looked for Argonaute proteins by annotating the conserved PIWI and PAZ domains in all 49 species. Domain alignments were retrieved from Pfam (PIWI accession: pfam02171; PAZ accession: pfam02170) (Mistry et al. 2021). Using HMMER, we built a profile for each multiple sequence alignment and searched the profiles against each (--*e*-value 10e^−6^). Only proteins with both domains annotated were considered Argonaute proteins and retained for phylogenetic analysis.

To annotate DICER proteins, we aligned the amino acid sequences against the bilaterian annotated set of Mukherjee and colleagues (2013) using blastp. In a second round of filtering, we retrieved the ribonuclease 3 domain alignment from Pfam (accession: pf14622.9), the only domain shared between all metazoan DICERs (Mukherjee et al. 2013); Scanning the sequences with HMMER (---value 10e^−6^), we selected only those containing the ribonucleases 3 domain. However, orthologs of the endoribonuclease DROSHA were possibly included among the annotated DICERs at this stage. Therefore, we downloaded the DROSHA orthologs from OrthoDB (reference: 9211at3208) (Zdobnov et al. 2021), and we built a custom data set with both DICER from Mukherjee and colleagues (2013) and DROSHA sequences. We locally aligned the set of putatively annotated DICER proteins against this data set. We retained proteins whose best five hits were all with DICER orthologs and discarded proteins with only DROSHA orthologs among the best five hits. No ambiguous results (i.e. proteins showing both DICER and DROSHA within the best five hits) were obtained.

The Argonaute and DICER phylogenetic trees were inferred from data sets comprising all annotated sequences from proteomes, genomes, and transcriptomes, with the addition of reference sequences chosen from SwissProt (supplementary table S1, Supplementary Material online) or the bilaterian annotated set for the DICER data set. Data sets were aligned with MAFFT v7.508 (Katoh and Standley 2013), using the options --maxiterate 1,000 --localpair. Uninformative columns were masked from the alignments using Gblocks (Castresana 2000), setting -b2 = (3 × number of sequences)/5 -b3 = 10 -b4 = 5 -b5 = a. Additionally, another masking tool, ClipKIT (Steenwyk et al. 2020), was used to assess the impact of the masking step on the phylogenetic analyses. Gblocks resulted in being more conservative than ClipKIT, masking most of the alignment columns (supplementary table S10, Supplementary Material online). However, the ML trees inferred from the two alignments showed no difference between each other regarding the presence of each Argonaute (i.e. miAGO, siAGO, PIWI1,2) or DICER protein in each lophotrochozoan species (supplementary table S10, Supplementary Material online). Therefore, only the alignment obtained with Gblocks was used for downstream analyses.

The ML trees were inferred with IQ-TREE (Nguyen et al. 2015) using the predefined protein mixture model LG + C20 + R4. To assess the robustness of the clades, we calculated the UFBoot with 1,000 bootstrap replicates (Hoang et al. 2018) and the SH-alrt test with 1,000 replicates (Guindon et al. 2010).

We tested whether DICER1 and miAGO proteins have experienced an intensified selection with HyPhy RELAX (Wertheim et al. 2015). We tagged all branches belonging to DICER1 and miAGO clades as foreground. All other branches belonging to DICER or AGO family clades were tagged as background. For some proteins, we were not able to retrieve the respective coding sequence, namely the *Saccostrea glomerata* miAGO and DICER1, *A. gambiae* DICER2, and *Trobolium castaneum* and *Brugia malayi* DICER1. Those proteins were removed during the selection analysis.

The completeness of proteomes, assemblies, and transcriptomes was evaluated with BUSCO v. 5.4.3 (Simão et al. 2015) using the Metazoa data set.

The annotation of the N (accession: pfam16486) and MID (accession: pfam16487) domains for Argonaute proteins and the Hel and PAZ domains for DICER proteins was made using HMMER, setting the *e*-value cutoff as the lowest *e*-value among outgroup sequences. The Hel and PAZ profiles were built from sequences downloaded from UniProt (Bateman et al. 2021): *D. melanogaster* DICER1 (accession: Q9VCU9), *D. melanogaster* DICER2 (accession: A1ZAW0), *N. vectensis* DICER1 (accession: U3MHS9), *Mytilus gallus* DICER (accession: A0A140H129), *C. elegans* DICER (accession: P34529), and *H. sapiens* DICER (accession: Q9UPY3).

### *N. geniculatus* sncRNA Library Sequencing and Analysis of sncRNA Libraries

Six specimens (three males and three females) of the nemertean *N. geniculatus* were sampled in June 2018 near Ushimado (Okayama prefecture, Japan). Animals were left in seawater and 7% MgCl_2_·H_2_O (1:1 ratio) for 15 s; gonads were then dissected in 7% MgCl_2_·H_2_O on ice and stored in RNAlater (Thermo Fisher Scientific Inc., Waltham, USA), following the manufacturer's instructions. Total RNA was extracted using a standard chloforom:TRI Reagent (Merck KGaA, Darmstadt, Germany) protocol, following manufacturer's instructions. The TruSeq Small RNA library kit (Illumina, San Diego, USA) was used to prepare six small RNA libraries that were sequenced on an Illumina HiSeq 2500 platform. Both library preparation and high-throughput sequencing were carried out at the Macrogen Inc. facility (Seoul, South Korea).

For this study, we sequenced the sncRNA pool from six samples of *N. geniculatus*. These libraries were analyzed alongside publicly available sncRNA libraries from five other species. The libraries were selected and downloaded from the Sequence Read Archive (SRA) provided by NCBI (supplementary table S11, Supplementary Material online). Where multiple samples from the same project were available, libraries were pooled together, to obtain a single fastq file for each species. Adapters and low-quality bases were removed from reads using Cutadapt v3.9.7 (Martin 2011), with the options -e 0.2 -O 5 --quality-cutoff 6 --discard-untrimmed. Trimmed reads were mapped on the reference genome using Bowtie (Langmead et al. 2009), allowing up to 100 multiple alignments (-m 100). The distribution of overlaps between reads was estimated using the python script *overlapping_reads.py* (https://github.com/ARTbio/tools-artbio/blob/master/tools/small_rna_signatures/overlapping_reads.py; Antoniewski 2014).

For *C. gigas*, *D. rerio*, *A. gambiae*, and *N. geniculatus*, we annotated miRNAs, siRNAs, and piRNAs. The tool miRDeep2 (Friedländer et al. 2012) was used to predict miRNAs, providing the already annotated miRNA set of that species from MiRGeneDB (Fromm et al. 2022) or miRBase (Kozomara et al. 2019), along with the annotated miRNAs of up to five closely related species. The novel predicted miRNAs were evaluated following the criteria established by Fromm et al. (2015) and discarding novel miRNAs with a STAR sequence coverage lower than five reads. Putative siRNA and piRNA pairs were predicted based on read overlaps (i.e. siRNA pairs overlap of the read length—2, piRNA pairs with an overlap of 10 nucleotides), calculated using *overlapping_reads.py*. Pairs of siRNAs and piRNAs were discarded when one of the paired small RNAs had a coverage lower than 5, the logarithmic ratio of the pair exceeded 1.5, or the pair mapped on a miRNA region. Differential expression of sncRNAs between somatic tissues and ovaries was tested with edgeR (Robinson et al. 2010) with a generalized linear model and a quasi-likelihood *F*-test (Lund et al. 2012). We chose a stringent *P*-value threshold of 0.001 to consider a small RNA as significantly differentially expressed. Novel miRNA genes in *N. geniculatus* were assigned to known or novel miRNA families. We locally aligned the preliminary structure of miRNA genes against the full set of MirGeneDB pre-miRNA using blastn. Novel genes were assigned to the best hit miRNA family only if they shared the same seed (supplementary table S7, Supplementary Material online). The remaining miRNA genes were clustered into novel miRNA families by blasting the pre-miRNAs against each other and comparing the seed sequence: miRNAs were clustered in the same family if they blasted against each other and the seed sequence differed by up to one nucleotide (supplementary table S8, Supplementary Material online).

## Supplementary Material

evae098_Supplementary_Data

## Data Availability

The data underlying this article are available in the article and in its online Supplementary material. Newly sequenced raw reads were uploaded to the SRA under the BioProject PRJNA942081. Python, bash, and R scripts written for the differential expression of miRNAs, piRNAs, and siRNAs have been uploaded on a GitHub repository (https://github.com/AlessandroFormaggioni/argo_Metazoa/tree/main/DE_pipeline). If further data supporting this article are required, they will be made available upon reasonable request to the corresponding author.
