# Supplementary material for: The Evolution and Characterization of the RNA Interference Pathways in Lophotrochozoa
Source: Genome Biol Evol. 2024 May 7;16(5):evae098. doi: 10.1093/gbe/evae098 (PMC11114477; doi:10.1093/gbe/evae098)
Supplement: evae098_Supplementary_Data [file evae098_supplementary_data.zip › Supplementary_figures.pdf]

# Supplementary figures

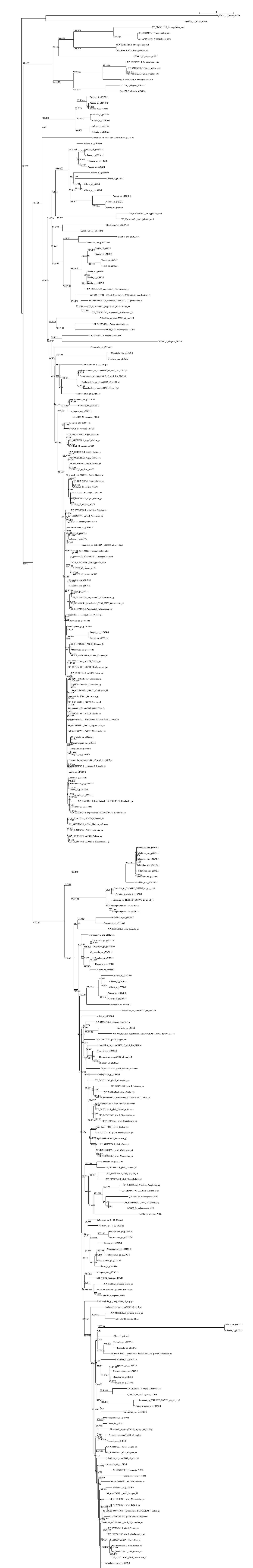

**Supplementary figure S1.** ML tree of the lophotrochozoan Argonauate proteins. For each node it is reported the SH-alrt and UFBoot value

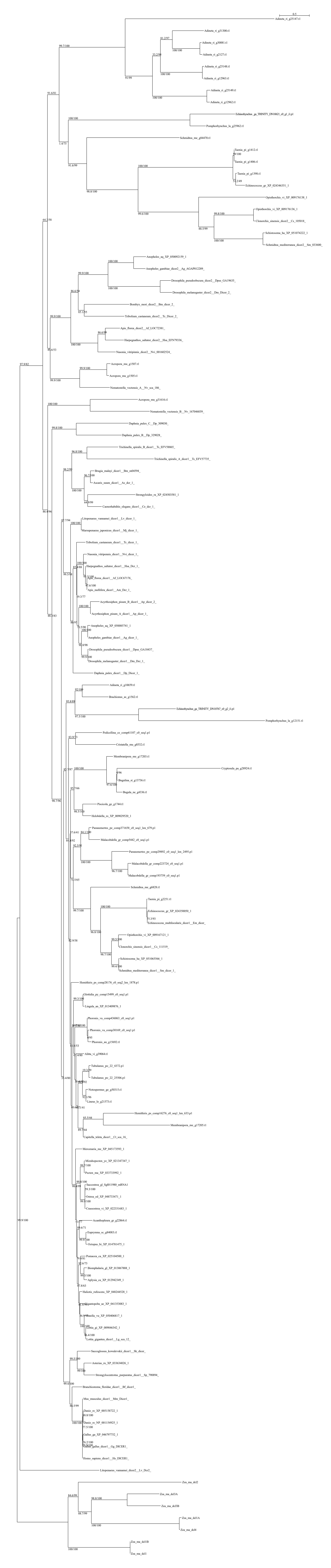

**Supplementary figure S2.** ML tree of the lophotrochozoan DICER proteins. For each node it is reported the SH-alrt and UFBoot value

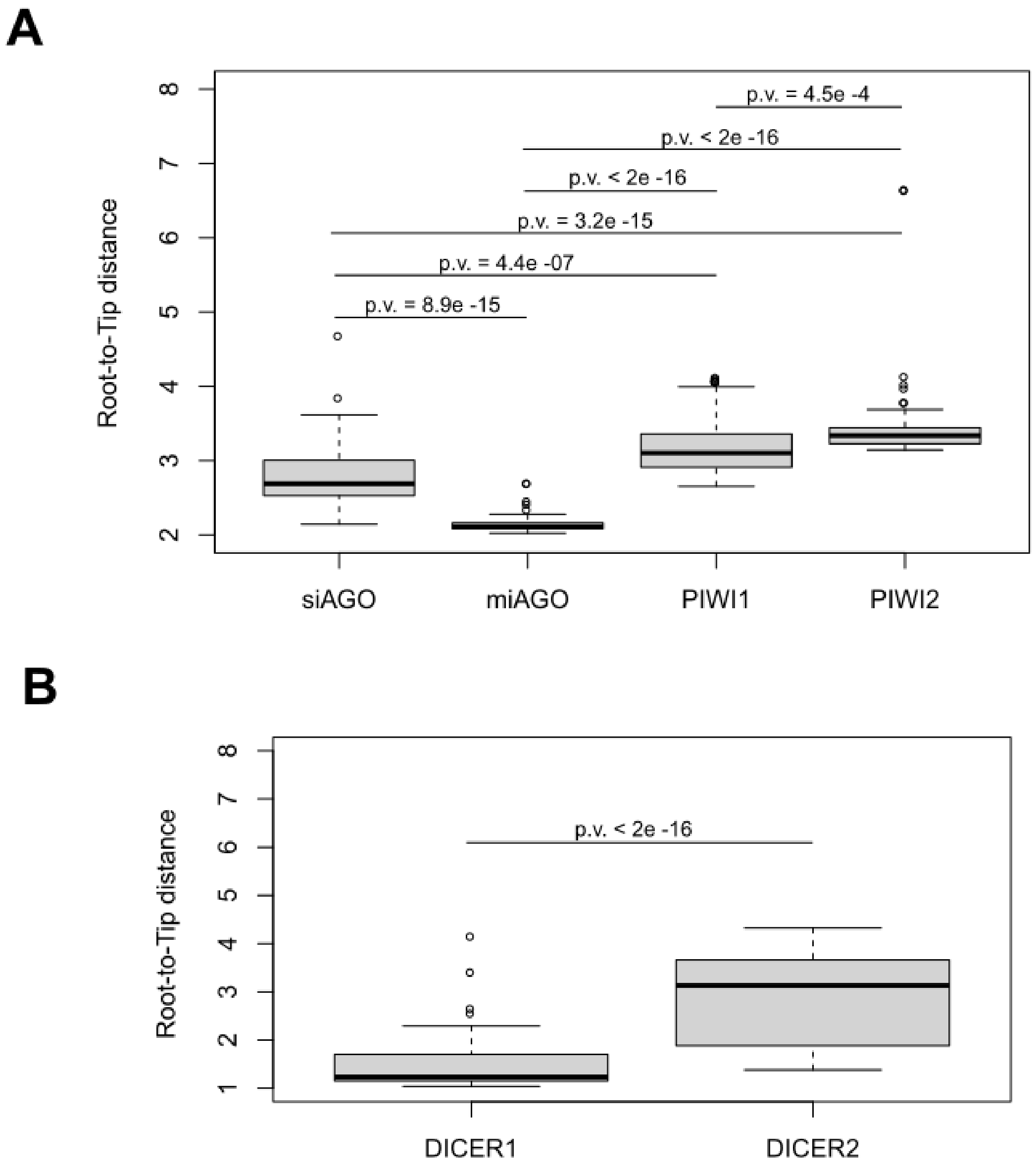


**Supplementary figure S3.** Root to tip distance distribution of Argonaute and DICER proteins. The distance of each tip from the root has been calculated by summing all the branch-lengths from the root to the tip. Significance between groups has been evaluated through an ANOVA test, followed by a pairwise t-test. All comparisons resulted significant. (A) Boxplot reporting the root to tip distance distribution of the four Argonaute proteins. Significance between groups was evaluated through an ANOVA test followed by a Tukey's HSD test. All comparisons resulted significant (B) Boxplot reporting the root to tip distance distribution of DICER proteins. According to the Student's t-test, the comparison between the two groups resulted significant.

**A**

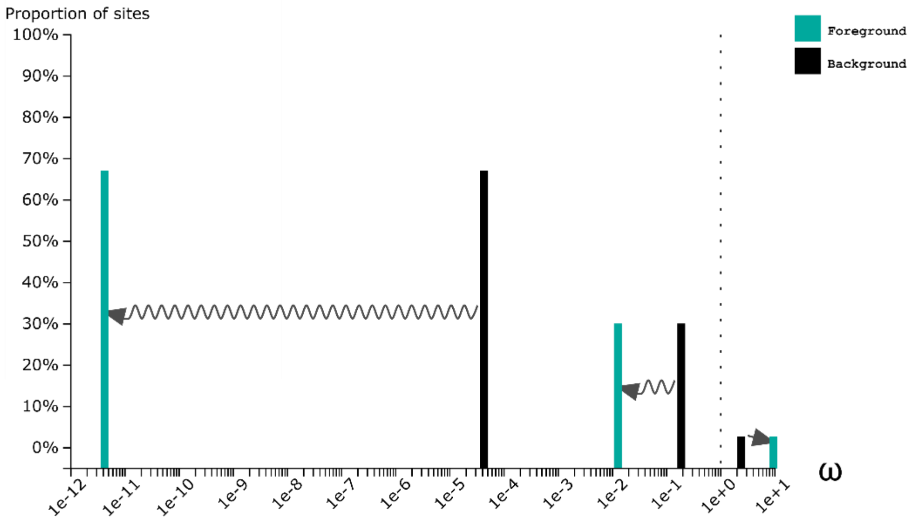


**B**

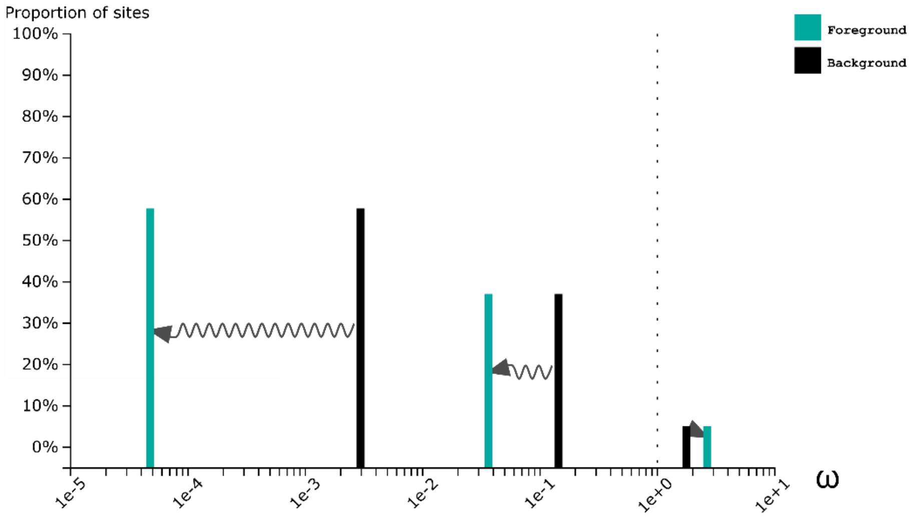


**Supplementary figure S4.** Sites distribution of the three ω classes under the RELAX alternative model. (A) Plot of the RELAX analysis on the AGO family**.** (B) Plot of the RELAX analysis on the DICER family

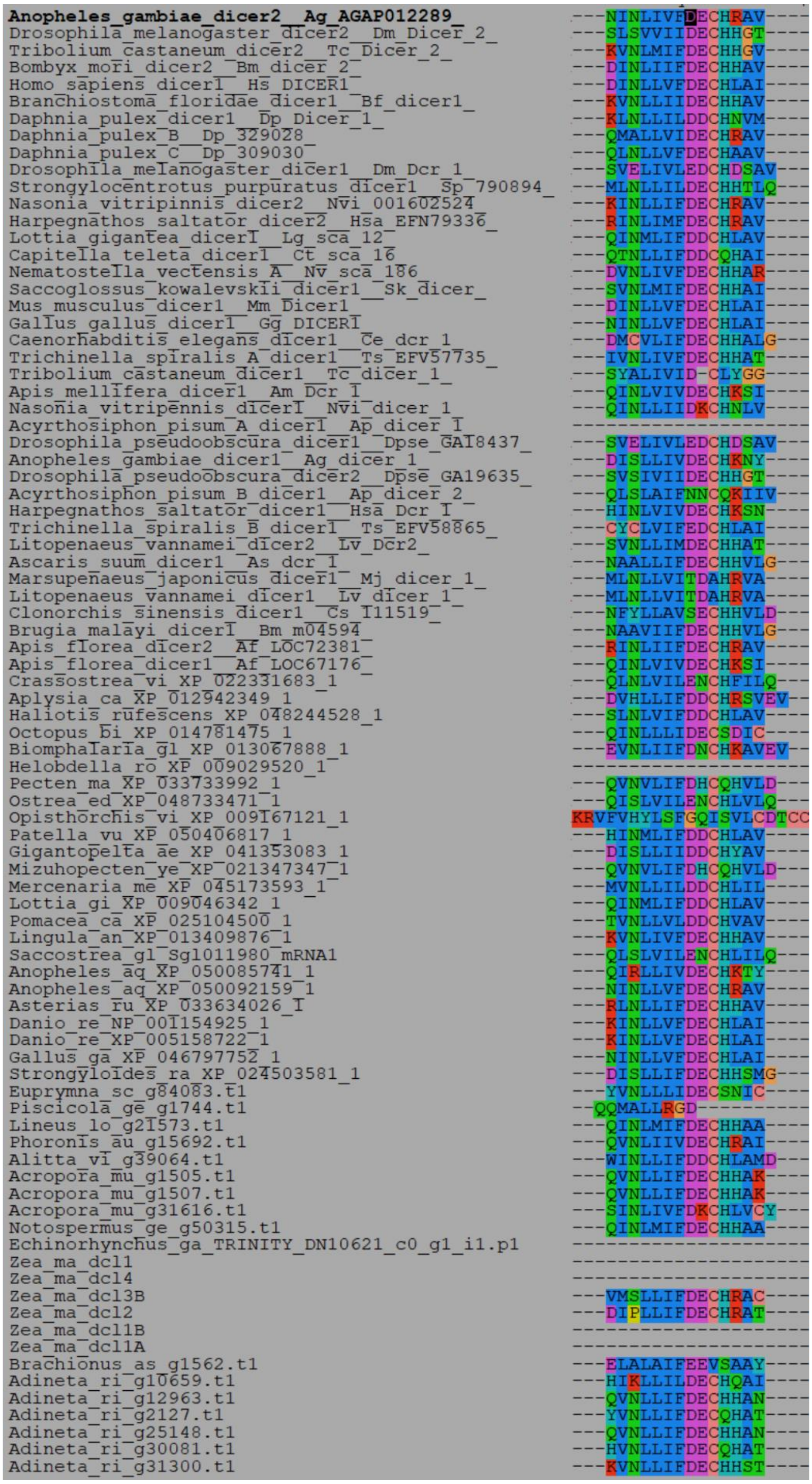

**Supplementary figure S5.** The conservation of the DECH box across Lophotrochozoa. Portion of the multiple sequence alignment of the Hel domain that comprehends the DECH box, which is a motif composed of four amino acids: Asp, Glu, Cys, His.

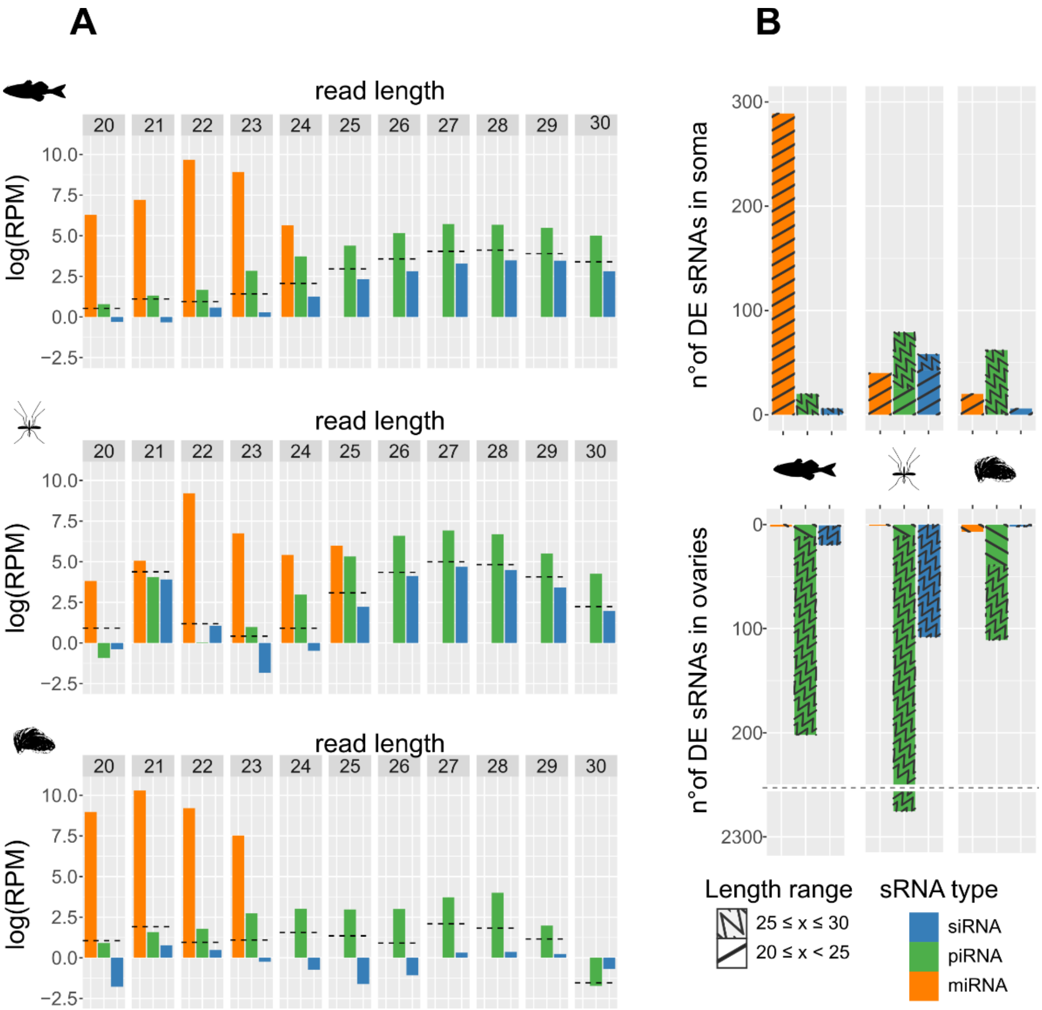


**Supplementary figure S6**. Expression of miRNAs (orange), piRNAs (green) and endo-siRNA (blue) at different lengths and in two tissues of *Danio rerio*, *Anopheles gambiae* and *Crassostrea gigas*. (A) Barplots report the expression of the three sRNA types expressed in reads per millions (RMP) on a logarithmic scale. sncRNAs were divided by length. Horizontal lines represent the background signal. To calculate the background signal we excluded reads annotated as miRNAs, siRNAs and piRNAs and computed the highest reads per million value (RPM) among the remaining overlap groups for each read length. We took that value as the background expression level of sRNA for that read length. We expect bona fide miRNAs, piRNAs and siRNAs to be more expressed than the background level in terms of RPM (B) The barplot reports the number of sRNAs significantly more expressed in the ovary against a somatic tissue and vice versa.

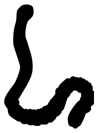

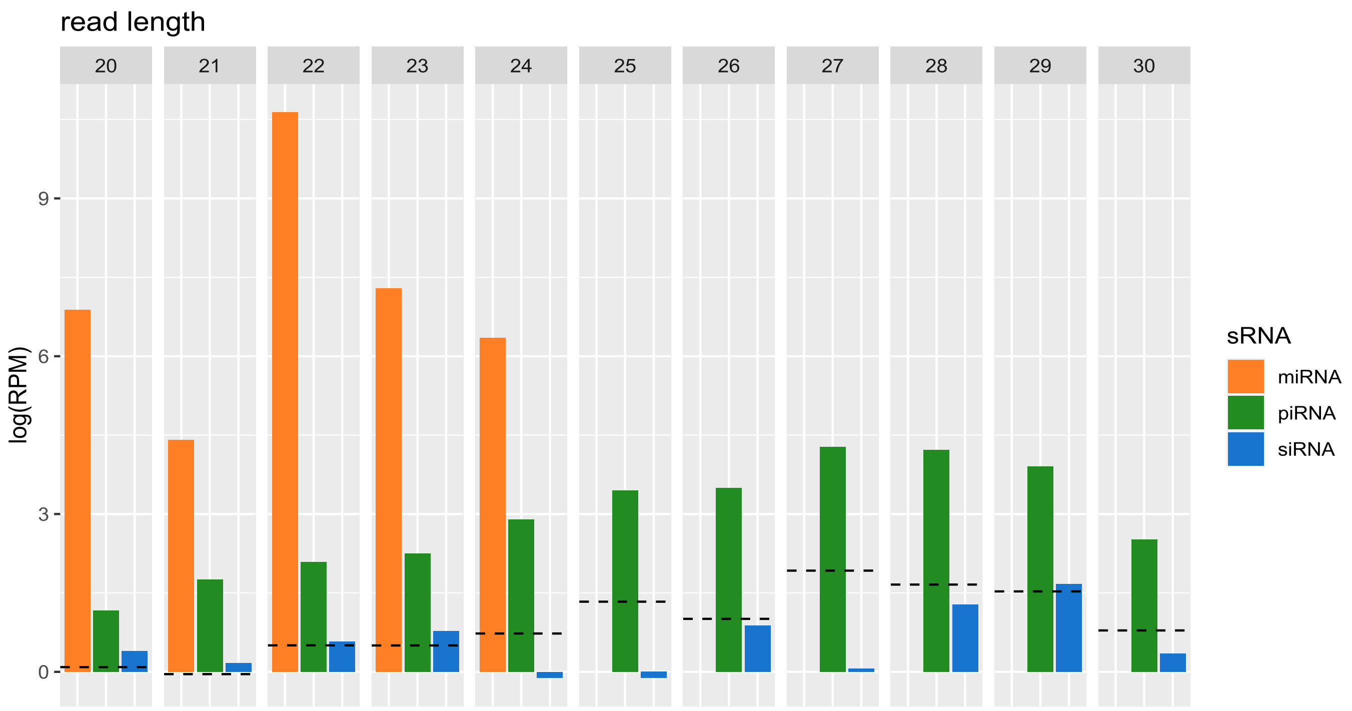


**Supplementary figure S7.** Expression of miRNAs (orange), piRNAs (green) and endo-siRNAs (blue) at different lengths of *Notospermus geniculatus*. Barplots report the expression of the three sRNA types expressed in reads per millions (RPM) in a logarithmic scale. To calculate the background signal (dashed line on the plots) we excluded the reads annotated as miRNAs, piRNAs and endo-siRNAs and computed the highest reads per million value among the remaining overlap groups for each read length. We took that value as the background expression level of sRNA for that read length. We expect that bona fide miRNAs, piRNAs and endo-siRNAs to be more expressed than the background level in terms of RPM.
